# Transcriptome-Wide Integrated Analysis of the *PgGT25-04* Gene in Controlling Ginsenoside Biosynthesis in *Panax ginseng*

**DOI:** 10.3390/plants12101980

**Published:** 2023-05-15

**Authors:** Lei Zhu, Jian Hu, Ruiqi Li, Chang Liu, Yang Jiang, Tao Liu, Mingming Liu, Mingzhu Zhao, Yi Wang, Kangyu Wang, Meiping Zhang

**Affiliations:** 1 College of Life Science, Jilin Agricultural University, Changchun 130118, China; zhulei0916@163.com (L.Z.); hujian2021420@163.com (J.H.); lrq972152210@163.com (R.L.); lchang1205@163.com (C.L.); jiangyangyang1031@163.com (Y.J.); liutao20210420@163.com (T.L.); lmm19971107@163.com (M.L.); mingzhuzhao@jlau.edu.cn (M.Z.); wanglaoshi0606@163.com (Y.W.); 2 Jilin Engineering Research Center Ginseng Genetic Resources Development and Utilization, Changchun 130118, China

**Keywords:** *Panax ginseng*, *PgGT25-04* gene, Trihelix transcription factors, ginsenoside biosynthesis, ABA stress

## Abstract

*Panax ginseng* is a valuable medicinal herb of the Araliaceae family with various pharmacological activities. The Trihelix transcription factors family is involved in growth and secondary metabolic processes in plants, but no studies have been reported on the involvement of *Trihelix* genes in secondary metabolic processes in ginseng. In this study, weighted co-expression network analysis, correlation analysis between *PgGTs* and ginsenosides and key enzyme genes, and interaction network analysis between *PgGTs* and key enzyme genes were used to screen out the *PgGT25-04* gene, which was negatively correlated with ginsenoside synthesis. Using ABA treatment of ginseng hair roots, *PgGT* genes were found to respond to ABA signals. Analysis of the sequence characteristics and expression pattern of the *PgGT25-04* gene in ginseng revealed that its expression is spatiotemporally specific. The interfering vector pBI121-PgGT25-04 containing the *PgGT25-04* gene was constructed, and the ginseng adventitious roots were transformed using the Agrobacterium-mediated method to obtain the pBI121-PgGT25-04 positive hairy root monocot line. The saponin contents of positive ginseng hair roots were measured by HPLC, and the changes in *PgGT25-04* and key enzyme genes in positive ginseng hair roots were detected via fluorescence quantitative RT-PCR. These results preliminarily identified the role of the *PgGT25-04* gene in the secondary metabolism of ginseng in Jilin to provide a theoretical basis for the study of Trihelix transcription factors in *Panax ginseng*.

## 1. Introduction

A transcription factor is a protein molecule with a specific structure that performs the function of regulating gene expression, also known as a trans-acting factor [1]. Transcription factors contain four key functional regions: the DNA binding region, the nuclear localization signal, the regulatory region, and the oligomerization site [2]. It can bind to specific DNA sequences (cis-acting elements) in the upstream promoter region of the target gene [3], and thus regulate the expression of the target gene in different tissues in plants and under different growth environments [4]. Transcription factors form a complex network that regulates gene expression during plant evolution, and they are also key regulators of multiple signaling pathways [5]. Different transcription factors such as MYB [6], bHLH [7], AP2/ERF [8], GRAS [9], NAC [10], Trihelix [11] and WRKY [12] play important regulatory roles in different plants.

The Trihelix transcription factor is a light-responsive protein, so named because of its typical triple-helix structure, and is also known as the GT factor, one of many plant transcription factors that function by binding to the GT element in its DNA-binding region [13]. The transcriptional activity of the GT factor is mainly regulated at the post-translational level [14]. The Trihelix family is divided into five subfamilies: the GT-1 family, the GT-2 family, the GTγ family, the SH4 family and the SIPI family [15]. Among these subfamilies, only the GT-2 transcription factor has two DNA-binding domains [16].

The Trihelix transcription factors family has an important role in plant growth and development, as well as stress [17]. *PrASIL1* expression in peony (*Paeonia suffruticosa*) is inversely correlated with lipid accumulation, and up-regulation of lipid biosynthesis genes by silencing *PrASIL1* resulted in significant increases in total lipids and several major fatty acids [18]. The *ShCIGT* gene in tomato (*Solanum lycopersicum*) responds to both abiotic stress and abscisic acid (ABA), and *ShCIGT* may enhance abiotic stress tolerance in tomato by interacting with *SnRK1* [19]. *GT-4* interacts with TEM2 protein to enhance salt stress tolerance in Arabidopsis (*Arabidopsis thaliana*) [20]. The wheat (*Triticum aestivum*) GT factor *TaGT2L1D* negatively regulates osmotic stress tolerance and plant development [21]. In strawberry (*Fragaria Ananassa* Duch.), expression of the FaGT-2-like gene was inhibited by high salt, drought, low temperature and ABA treatment [22]. The *BnSIP1-1* gene in oilseed rape (*Brassica chinensis*) responds to ABA signaling and salt stress. Overexpression of *BnSIP1-1* allows seeds to germinate even under salt stress and ABA treatment, and *BnSIP1-1* was found to reduce the sensitivity of transgenic seedlings to both osmotic stress and ABA treatment, and *BnSIP1-1* plays different roles in ABA synthesis and ABA signaling pathways and salt stress [23]. Overexpression of the apple (*Malus domestica*) *MdSIP1-2* gene promotes lateral root development and improves abscisic acid (ABA) sensitivity, and *MdSIP1-2* plays an important role in root development, ABA synthesis and tolerance to salt and drought stress [24]. These findings suggest that members of the *Trihelix* family of transcription factors play an important role in the biotic and abiotic regulation of growth and development, response to ABA signaling, salt resistance and other signaling pathways in plants.

Abscisic acid (ABA) affects plant growth and development in response to biotic and abiotic stresses, and also plays a crucial role in plant secondary metabolism [25]. ABA promotes the synthesis of anthocyanins in grape (*Vitis vinifera*) cell cultures [26]. ABA induces the synthesis of α-tocopherol and THC in the MEP pathway and down-regulates the biosynthesis of terpene metabolites in the MEP and MVA pathways in cannabis (*Cannabis sativa*) [27]. ABA significantly increased the content and yield of four tanshinones in the hairy roots of *Salvia miltiorrhiza* [28]. ABA regulates the production of 3-hydroxy-3-methylglutaryl CoA reductase gene promoter and ginsenosides in *Panax quinquefolius* ginseng hairy root cultures [29].

Ginseng (*Panax ginseng*) is a valuable medicine and is known as the king of all herbs [30]. Ginseng has a history lasting over 2000 years and is widely used in medicine, dietary supplements and other applications [31]. Ginseng contains a wide range of active ingredients, with ginsenosides being the main active ingredient in ginseng and likewise a secondary metabolite in ginseng [32,33,34]. The ginsenoside synthesis pathway is divided into the mevalonate (MVA) pathway and the methyl-erythritol-4-phosphate (MEP) pathway. More than 30 key enzyme genes of the ginsenoside biosynthesis pathway have been identified from plants and microorganisms of the genus Ginseng [35], examples of which include farnesyl pyrophosphate synthase (*FPS*), squalene epoxidase (*SQE*), oxidative squalene cyclase (*OSC*), cytochrome P450s (*CYP450*), UDP-glycosyltransferases (*UGT*), etc. Many transcription factors have been identified in ginseng, such as NF-Y [36], MYB [37], bHLH [38], AP2/ERF [39], GRAS [40], NAC [41] and WRKY [42], and most of them respond to abiotic stresses such as salt stress and methyl jasmonate signaling, while some are also involved in ginseng secondary metabolic processes [43]. In contrast, studies on the involvement of *Trihelix* genes in ginseng secondary metabolism have not been reported.

In this study, *PgGT25-04* is closely related to ginsenoside synthesis, which was selected from the *PgGT* genes by means of weighted gene co-expression network analysis (WGCNA) and correlation analysis as well as reciprocal network analysis, and the ginsenoside content of the key enzyme genes and *PgGT* genes was verified by treating ginseng root with ABA. The sequence characteristics and expression patterns of the *PgGT25-04* gene were then analyzed, and an RNAi silencing vector for the *PgGT25-04* gene was constructed. Ginseng adventitious roots were transformed via the Agrobacterium-mediated method, and the expression of ginsenosides and key enzyme genes was measured after obtaining hairy roots. The *PgGT25-04* gene was initially verified to be involved in the biosynthesis of ginsenosides, which laid the foundation for the study of *PgGT* genes in ginseng and also provided a theoretical basis for the study of secondary metabolic processes in ginseng.

## 2. Results

### 2.1. Weighted Gene Co-Expression Network Analysis (WGCNA) of PgGT Genes

**Figure 1:**
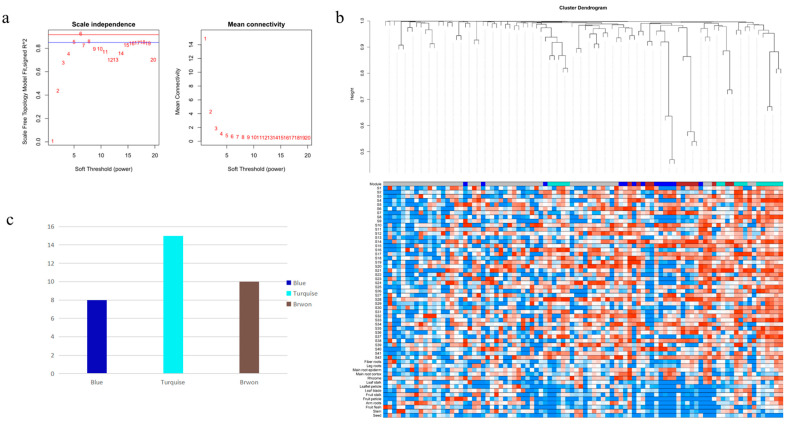
WGCNA of the *PgGT* genes. (**a**) Determination of soft threshold. (**b**) Gene topological overlapping clustering tree. The modules of the co-expressed genes are shown with differently colored bars at the bottom of the dendrogram. (**c**) Number of genes in 3 modules.

**Figure 2:**
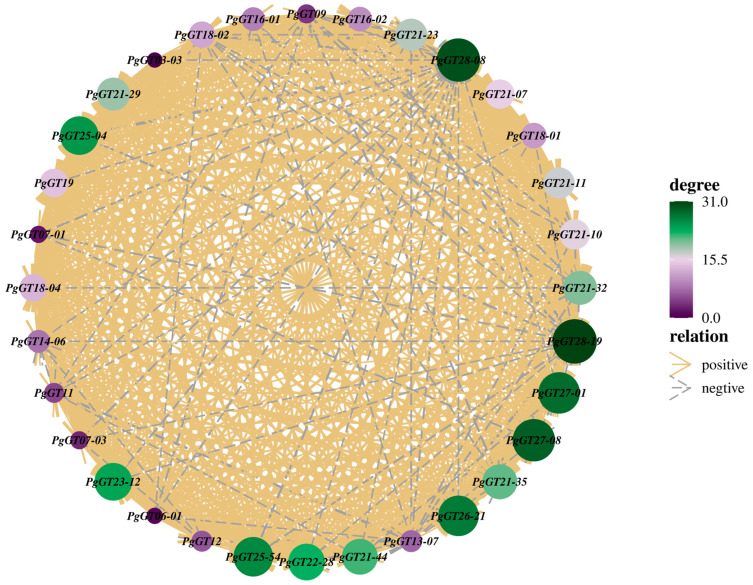
Network diagram of correlation between key *PgGT* genes. Each circle represents a *PgGT* gene. Solid yellow lines between genes indicate the presence of synergistic interactions between genes. The blue dashed lines between genes indicate the presence of antagonistic effects between genes.

We used the pick-Soft-Threshold function of the WGCNA package in R to calculate the weight values and determine the best soft threshold for these data. When a soft threshold value *β* = 6 was chosen, its scale-free network fit index *R*^2^ > 0.85 and its average connectivity converged to 0. Therefore, a soft threshold value *β* = 6 was determined to construct a weighted co-expression network (Figure 1a). After determining the soft threshold, a cluster dendrogram was generated based on topological overlap using gene averaging chain hierarchical clustering, which revealed that the modules of co-expressed genes could be divided into three modules at the bottom of the dendrogram (Figure 1b). The turquoise module has the highest number of genes, with 15 key genes; the blue module has the lowest number of genes, with 8 (Figure 1c). Genes in the three modules are shown in Table S1, which have synergistic effects in the same module. Network analysis of these 33 genes showed that most of the genes had synergistic effects, while only a few genes had antagonistic effects (Figure 2).

### 2.2. Identification of Genes Associated with Ginsenoside Biosynthesis

**Figure 3:**
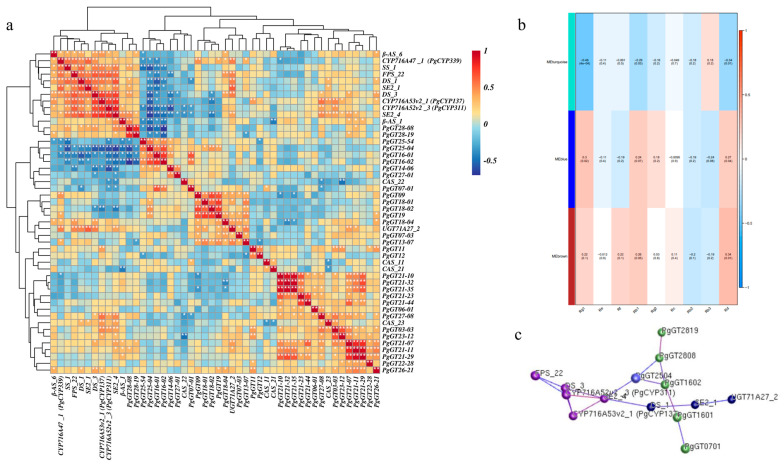
Identification of genes associated with ginsenoside biosynthesis. (**a**) Heat map of the correlation between *PgGT* genes and key enzyme genes of ginsenoside synthesis by clustering. (**b**) Correlation heat map between three gene modules and ginsenosides. (**c**) Analysis of interaction network between three modules of genes and key enzyme genes. “*” as significant at *p* ≤ 0.05, “**” as significant at *p* ≤ 0.01.

Correlations were calculated between the expression of these 33 *PgGT* genes and key enzyme genes, and a heat map was plotted, showing that the *PgGT25-04* gene was most significantly associated with key enzyme genes among these core genes (Figure 3a). To determine the relationship between different gene modules and ginsenoside content, correlation coefficients between all gene modules and individual ginsenosides (Rg1, Re, Rf, Rb1, Rg2, Rc, Rb2, Rb3 and Rd) were calculated and correlation analyses were performed, with the turquoise module correlation coefficient r values at *p* < 0.01 being the most correlated with the expression of ginsenoside Rg1, and therefore the genes within the turquoise module were highlighted for analysis, while the most significant gene associated with the key enzyme gene, *PgGT25-04*, was also present in this module (Figure 3b). Finally, the 33 genes and key enzyme genes were mapped for interaction network analysis. When the *p*-value was 1.00 × 10^−5^, we found that the *PgGT07-01*, *PgGT16-01*, *PgGT16-02*, *PgGT25-04*, *PgGT28-08* and *PgGT28-19* genes are closely associated with key enzymes (Figure 3c). However, the *PgGT25-04* gene directly and closely interacts with key enzyme genes, and is also a key gene in the turquoise module. These results indicate that *PgGT25-04* is a negatively regulated transcription factor closely related to key enzyme genes, and we speculate that it affects ginsenoside biosynthesis. Therefore, *PgGT25-04* was selected as the key research object for the subsequent functional identification, and the subsequent functional research was conducted.

### 2.3. ABA Regulates the Expression of PgGT Genes

**Figure 4:**
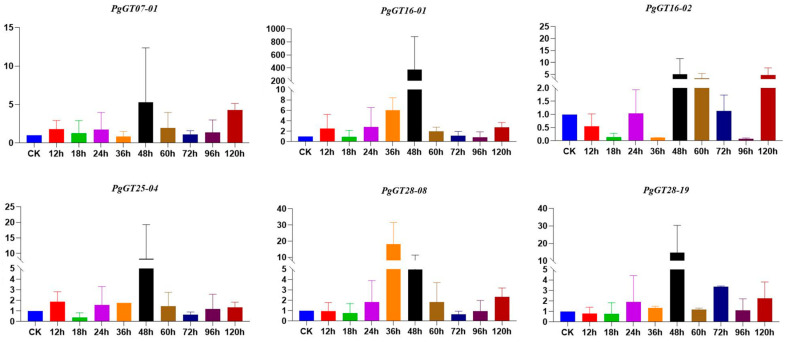
Expression levels of *PgGT* genes under ABA stress. The X axis represents the duration of ABA treatment of ginseng hairy roots. The Y axis represents the relative expression level of *PgGTs* in ginseng hairy roots.

The total RNA of ginseng roots treated with ABA at different times was extracted and reverse transcribed into cDNA. After fluorescence RT-PCR, we found that the expression of six *PgGT* genes, which are closely related to the key enzyme genes, was changed. Five of these genes reached their highest expression at 48 h. The expression of *PgGT16-01* increased 360-fold at 48 h. All six *PgGTs* were up-regulated at 120 h compared to the control. We found that the expression of most of the *PgGT* genes showed an increase after ABA treatment (Figure 4). We also found that the expression of key enzyme genes appeared to be reduced at multiple time points and that ABA inhibited the expression of key enzyme genes. In particular, the *PgUGT* gene, which was up-regulated except at 120 h, was down-regulated for the rest of the treatment time (Figure S1). Interestingly, after a short time, ABA treatment promoted the release of ginsenosides—the ginsenosides Rg1, Rd, PPT and PPD were all increased at 12 h compared to 0 h. However, ginsenoside Rd was the lowest at 48 h, and the remaining three ginsenosides were all decreased at this time (Figure S2).

### 2.4. Sequence Characterization and Expression Pattern Analysis of PgGT25-04

**Figure 5:**
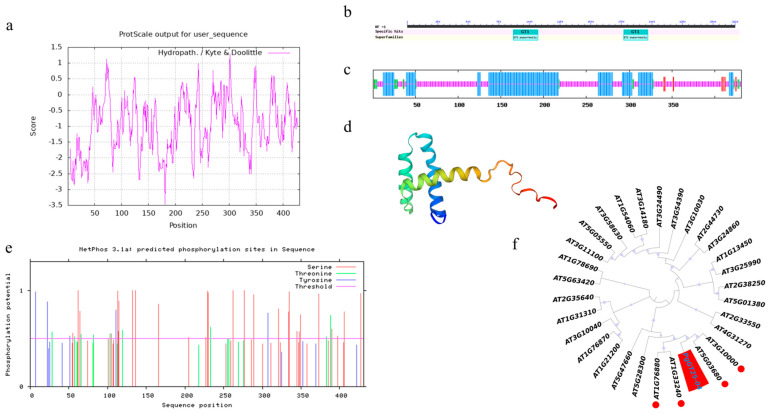
Sequence characterization analysis of *PgGT25-04*. (**a**) *PgGT25-04* hydrophilicity analysis. (**b**) Conserved domain of PgGT25-04 amino acid sequence. (**c**) Prediction of secondary structure of PgGT25-04 protein. The α helix is depicted in red, and the β−corner is depicted in green, the extension chain is depicted in red and the random crimp is depicted in purple. (**d**) Prediction of tertiary structure of the PgGT25-04 protein. (**e**) Phosphorylation sites of the PgGT25-04 protein. (**f**) Phylogenetic tree of amino acid sequences of PgGT25-04 and members of the Arabidopsis Trihelix transcription factor family. Arabidopsis genes marked with red circles belong to the GT-2 subfamily.

The *PgGT25-04* gene from ginseng has a total length of 2677 bp and an open reading frame of 1296 bp, and the PgGT25-04 protein encodes a total of 431 amino acids. The protein is relatively unstable in an aqueous environment and has a high affinity for water molecules and is readily soluble in water (Figure 5a). Post-translational modification is closely related to protein stability. The *PgGT25-04* gene has a GT-1 conserved domain at the N terminal and C terminal, respectively. Structural proof that it is a member of the GT-2 subfamily is provided in Figure 5b. Online software was used to predict the secondary structure of PgGT25-04 protein. The α helix accounted for 37.59%, the random curling structure accounted for 55.45%, the β-angle accounted for 4.18% and its extended chain accounted for 2.78%, which was mainly composed of spiral and random curling. It has a typical triple helix structure (Figure 5c). The tertiary structure of this gene was predicted and the tertiary structure diagram model of PgGT25-04 protein was constructed. It can be clearly observed that its spatial structure is a triple helix structure (Figure 5d). Prediction analysis of 17 kinases, including cdc2, cdk5 and p38MAPK, by analyzing phosphorylation sites using online software revealed that the gene fragment contained multiple phosphorylation sites, 41 of which had a score value greater than 0.5 and proved to be positive. Through TMHMM online analysis, serine 42 sites, threonine 18 sites and tyrosine 12 sites proved that *PgGT25-04* is not a transmembrane protein (Figure 5e). Through phylogenetic tree analysis of *PgGT25-04* gene and Arabidopsis Trihelix transcription factor family members in Jilin ginseng, we can see that *PgGT25-04* is closely related to GT-2 subfamily members in *Arabidopsis thaliana* (Figure 5f). 

**Figure 6:**
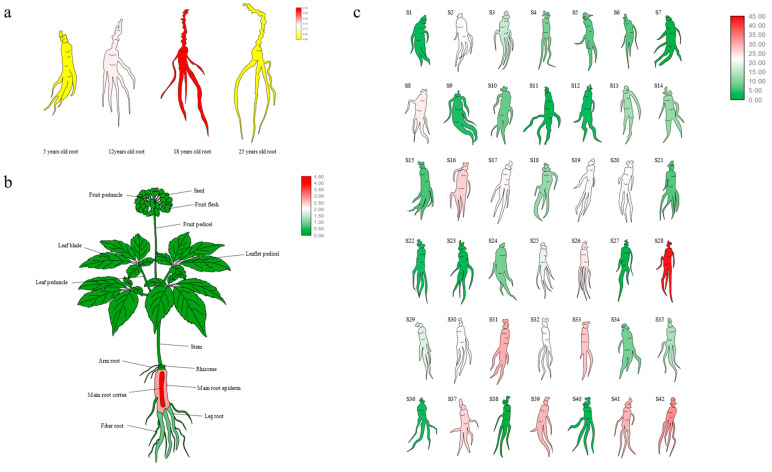
Heatmaps analysis of spatiotemporal expression patterns of the *PgGT25-04* gene in *Panax ginseng*. (**a**) The *PgGT25-04* gene’s expression in the 4 different ages of ginseng roots. (**b**) The *PgGT25-04* gene’s expression in the 14 different tissues of 4-year-old ginseng. (**c**) The *PgGT25-04* gene’s expression in the 42 farm cultivars of 4-year-old ginseng roots.

The expression of *PgGT25-04* increased with the increase in root age below a root age of 18 years old (Figure 6a). In 14 different tissues, the expression of the *PgGT25-04* gene was the highest in the main root cortex, followed by the main root epiderm (Figure 6b). The *PgGT25-04* gene was expressed in 42 different farm cultivars, and the expression situation was different in different farm cultivars, with the expression level being the highest in S28 (Figure 6c).

### 2.5. Construction of the PgGT25-04 Gene Silencing Vector

**Figure 7:**
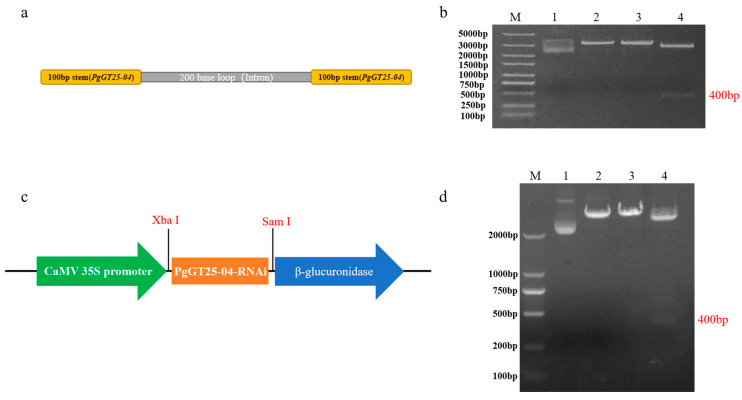
Construction of the *PgGT25-04* gene silencing vector. (**a**) PgGT25-04-RNAi sequence. (**b**) PgGT25-04-RNAi linked clone vector enzyme digestion verification. M: DL5000 marker; 2–3: single enzyme digestion; 4: double enzyme digestion. (**c**) pBI121-PgGT25-04 expression vector. (**d**) *PgGT25-04* interference vector enzyme digestion verification diagram. M: DL2000 marker; 2–3: single enzyme digestion; 4: double enzyme digestion.

The PgGT25-04-RNAi silencing sequence was designed (Figure 7a), and the PgGT25-04-RNAi interfering fragment was amplified by PCR according to the primer specificity, and then ligated into the cloning vector and double cleaved by two restriction endonucleases, *Sma*I and *Xba*I (Figure 7b). The RNAi interfering fragment was ligated to the expression vector pBI121 (Figure 7c). This was verified by double digestion with both *Sma*I and *Xba*I enzymes and the band was clearly visible (Figure 7d), demonstrating the successful construction of the recombinant plasmid pBI121-PgGT25-04.

### 2.6. Genetic Transformation of Ginseng Explants

**Figure 8:**
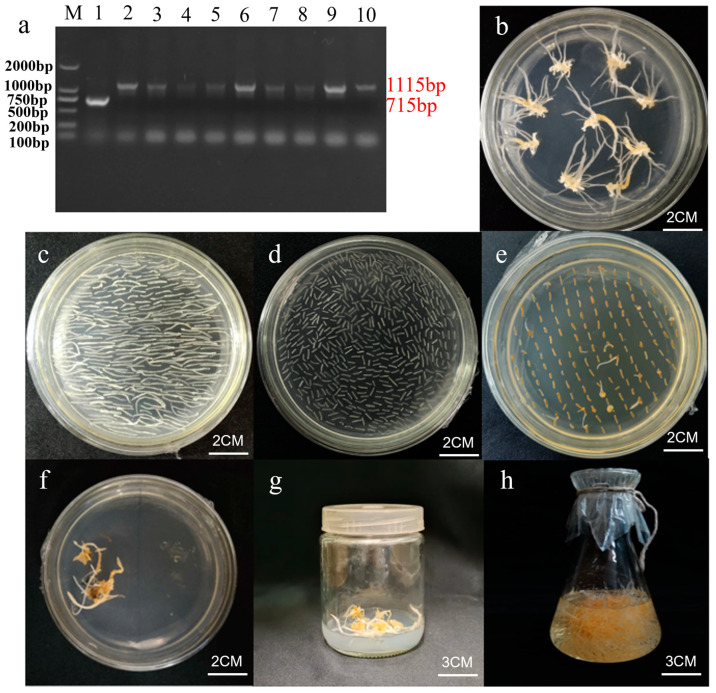
Genetic transformation of ginseng explants. (**a**) PCR electrophoresis map of C58C1 bacteria containing pBI121-PgGT25-04 recombinant plasmid. M: DL2000 marker; 1: empty vector control, 2–10: the result of PCR amplification of bacterial liquid. (**b**) Experimental materials with adventitious roots. (**c**) Preculture of adventitious roots. (**d**,**e**) Culture after infecting adventitious roots with bacterial solution. (**f**,**g**) The hairy root was cultured in solid state. (**h**) Single-root system of hairy root cultured by liquid propagation.

The *PgGT25-04* RNAi interferon vector pBI121-PgGT25-04 was successfully transformed into an *Agrobacterium tumefaciens* C58C1 receptor state via freeze–thawing (Figure 8a). The ginseng explants were transformed using the Agrobacterium-mediated method and cultured in the dark for a period of time to grow ginseng hairy roots, which were then cultured in succession when they reached 1 cm in length and eventually expanded in 250 mL shake flasks (Figure 8b–h).

### 2.7. Validation of Transgenic Hairy Root Monocots

The *RolC* gene fragment (500 bp) specific to *Agrobacterium tumefaciens* in each single hair root was examined. Using water as a negative control, and using positive plants containing empty bacterium C58C1 as a positive control, PCR detection of genomic DNA was carried out to detect the length of the *PgGT25-04* RNAi interference fragment inserted by two enzyme restriction sites of the pBI121 vector, which was 1115 bp. That of the *PgGT25-04* RNAi interference fragment was 400 bp. PCR was performed using the genomic DNA of the control group and different hair roots as templates. Results bands can be clearly observed (Figure S3), and it can be seen from the results that seven single-root systems of *PgGT25-04* RNAi interfering with transgenic ginseng hair roots were successfully obtained. Positive materials were retained, and a large number of single-root systems of positive materials were propagated for subsequent experiments.

### 2.8. Detection of Gene Expression and Ginsenoside Content in Positive Hairy Roots

**Figure 9:**
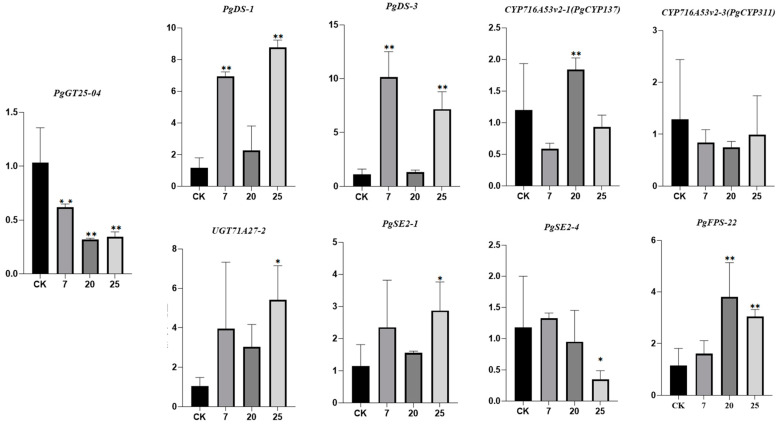
Expression levels of the *PgGT25-04* gene and key enzyme genes in ginsenoside synthesis in positive hairy roots. The *X*-axis indicates the number of ginseng hairlike roots. CK is the control, while 7, 20 and 25 are the numbers of ginseng positive hairlike roots. The *Y*-axis represents the relative expression of genes. “*” as significant at *p* ≤ 0.05, “**” as significant at *p* ≤ 0.01.

**Figure 10:**
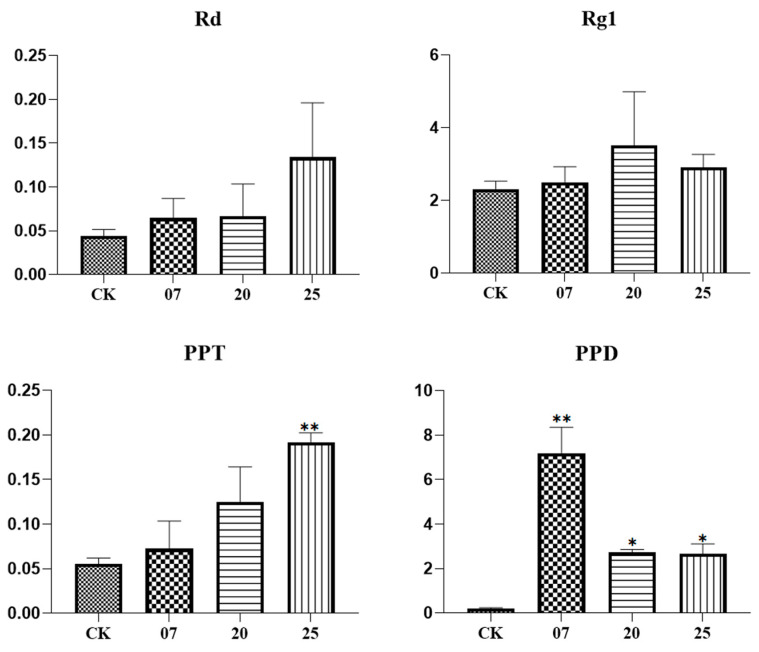
The contents of ginsenosides in positive hair roots. The *X*-axis indicates the number of ginseng hairlike roots. CK is the control, while 7, 20 and 25 are the numbers of ginseng positive hairlike roots. The *Y*-axis represents the relative content of ginsenosides. “*” as significant at *p* ≤ 0.05, “**” as significant at *p* ≤ 0.01.

Three transgenic ginseng hair roots (07, 20, 25) were selected for fluorescence quantitative PCR detection. The expression levels of the *PgGT25-04* gene and eight key enzyme genes related to the ginsenoside synthesis pathway were detected. After the interference of the *PgGT25-04* gene with RNAi, the expression levels of the key enzyme genes *DS-1*, *DS-3*, *FPS_22*, *CYP716A53v2_1* (*PgCYP137*), *SE2-1* and *UGT71A27_2* were significantly increased. The expression levels of *CYP716A52v2_3* (*PgCYP311*) and *SE2-4* showed a decreasing trend (Figure 9). Therefore, interference with *PgGT25-04* gene expression may reduce the production of some hindrance substances in the ginsenoside biosynthesis pathway, and thus increase the expression of key enzyme genes. Compared with the control group, the saponin content in the hair roots of transgenic ginseng was increased in the single-root system of the three transgenic plants (Figure 10). The results indicate that RNAi expression of *PgGT25-04* interfered with the biosynthesis of the above ginsenosides. It can be concluded that the *PgGT25-04* gene has a significant negative correlation with ginsenoside biosynthesis.

## 3. Discussion

Studies have shown that triple helix transcription factors can regulate the synthesis of secondary metabolites. In patchouli (*Pogostemon cablin*), *PatGT-1* negatively regulates the biosynthesis of fulicol by inhibiting genes in the pathway [44]. The secondary metabolism of Trihelix transcription factor in ginseng has not been studied deeply. The main active substance of ginseng is ginsenoside, and the overexpression of *PgGRAS68-01* in ginseng inhibits ginsenoside synthesis [43]. Genome-wide analysis of MADS-box gene revealed that *PgMADS41* and *PgMADS44* regulate root development and promote ginsenoside synthesis [45]. Our laboratory has also previously identified the Trihelix transcription factor family, which provides data support for our subsequent research. The WGCNA method is an efficient and fast mining method that computes transcriptome expression data, then searches for co-expressed gene modules through transcript data and classifies transcripts, and finally obtains gene co-expression modules with biological significance [46]. In order to explore whether members of the Trihelix transcription factor family participate in the regulation of secondary metabolism in Jilin ginseng, we performed WGCNA on the expression levels of 218 identified *PgGTs* transcripts in 42 farm cultivars and 14 different tissues. By calculating the connectivity of genes in the module, three key modules are selected: turquoise, blue and brown. WGCNA was also used to screen out age-related gene modules in pine (*Pinus species*) [47]. The correlation analysis between the genes of these three modules and ginsenoside expression showed that the turquoise module had the most significant correlation with ginsenoside, and showed a negative correlation. Then, correlation analysis was conducted between the genes in the three modules and ginsenoside gene expression levels, and it was found that the *PgGT25-04* gene was significantly negatively correlated with most key enzyme genes in ginsenoside synthesis. The interaction network analysis between the genes of the three modules and the key enzyme genes showed that only the *PgGT25-04* gene was still closely related to the key enzyme genes under the condition of a decreasing *p* value. Interestingly, the *PgGT25-04* gene is present in the blue-green module of the gene. This further indicated that *PgGT25-04* gene was involved in ginsenoside synthesis and inhibited its biosynthesis.

ABA is closely related to secondary metabolites. In *Glycyrrhiza uralensis*, the *GuPYL4*, *GuPYL5*, *GuPYL8* and *GuPYL9* genes were significantly up-regulated under ABA stress [48]. Tobacco (*Nicotiana tabacum*) *NtPYLs* play an important role in seed development, germination and response to ABA [49]. In *Zea mays*, *ZmPYL3* and *ZmPP2C16* proteins of maize interact in response to exogenous ABA induction [50]. In order to verify the involvement of *PgGT* genes in secondary metabolism, we treated ginseng hairy roots with ABA, and found that the expression of most *PgGT* genes was up-regulated, and the expressions of most key enzyme genes and ginsenoside contents were down-regulated, further proving that *PgGT* genes negatively regulate ginsenoside synthesis.

We analyzed the sequence characteristics and expression patterns of the *PgGT25-04* gene, and found that the *PgGT25-04* gene has typical transcription factor model characteristics, and its expression in ginseng is specific in both time and space. RNA interference (RNAi) refers to the highly conserved phenomenon of highly efficient and specific degradation of homologous mRNA induced by double-stranded RNA (dsRNA) in the course of evolution [51]. RNAi interference was applied in wheat (*Triticum aestivum*) [52,53], American ginseng (*Panax quinquefolius*) [54,55] and Arabidopsis (*Arabidopsis thaliana*) [56]. Since *PgGT25-04* is a negatively regulated transcription factor, we inhibited the expression of *PgGT25-04* via RNAi. We constructed a silent expression vector of the *PgGT25-04* gene, transformed ginseng adventitious roots using the Agrobacterium-mediated method, and detected ginsenoside content and expression levels of the *PgGT25-04* gene and key enzyme genes by means of HPLC and fluorescence quantitative PCR. When the *PgGT25-04* gene was interfered by using RNAi, the expression level of the *PgGT25-04* gene decreased, while the expression levels of the *FPS22*, *SE2-1*, *DS-1*, *DS-3* and *UGT71A27-2* key enzyme genes increased significantly. Among the other three key enzyme genes, only the *PgCYP311* gene was down-regulated, and the other two key enzyme genes were also up-regulated in the ginseng hairy roots. Ginsenoside PPT and ginsenoside PPD saponins were significantly increased. The accumulation of ginsenoside Rg1 and ginsenoside Rd also increased, which also verified that the increase in key enzyme genes in the ginsenoside synthesis pathway would affect the ginsenoside content. The decrease in *PgGT25-04* gene expression promoted the expression of key enzyme genes in the ginsenoside synthesis pathway and affected the accumulation of ginsenoside. These results verified the accuracy of previous bioinformatics predictions and provided valuable data for the study of Trihelix transcription factors in ginseng. The interaction between the GT factor and GT element is related to the complex transcriptional regulation of many plant genes. Since the mechanism of *PgGTs* in ginseng has not been fully elucidated, it is still uncertain as to whether the target gene bound by *PgGT25-04* is a structural protein or a transcription factor of the same or different species, which requires further study.

## 4. Materials and Methods

### 4.1. Plant Materials and Databases

The ginseng database used in this study was the ginseng Unigenes database established by Jilin engineering Research Center Ginseng Genetic Resources Development and Utilization [57]. The *PgGT* gene family was obtained from the previous identification at our laboratory. The plant material used in this study was ginseng adventitious root material, which was induced in our laboratory, and cultured and propagated in B5 medium. Ginseng hair roots treated with abscisic acid were single ginseng hair roots independently cultured and propagated in 1/2 MS medium. The bacteria and vectors used in this study were kept in the laboratory.

### 4.2. Weighted Gene Co-Expression Network Analysis (WGCNA) of PgGT Genes

We used R 4.1.2 for the WGCNA [46] expression levels of *PgGTs* in 42 farm cultivars and 14 different tissues. The soft threshold power was calculated using the pick-Soft-Threshold function. According to the approximate scale-free topology criterion, the most suitable power was selected to form the network. *PgGT* gene correlations and soft thresholds were calculated using a Pearson correlation matrix and network topology analysis. An appropriate soft threshold was selected to transform the original matrix, and then the adjacency matrix was transformed into a topological overlapping matrix, and the dynamic cutting method was used to cluster genes and divide modules. The merging threshold between similar modules was greater than 0.85. The expression patterns of module genes in each sample were displayed with module eigenvalues, and the heat map of sample expression patterns was drawn. The *PgGT* genes were selected from the module for correlation analysis and the co-expression interaction network map was drawn. Specific modules significantly correlated with ginsenoside Rg1, Re, Rf, Rb1, Rg2, Rc, Rd, Rb2 and Rb3 contents were determined via module feature vector gene analysis, and corresponding modules were selected for further study.

### 4.3. Correlation Analysis between PgGT Gene Expression Level and Key Enzyme Gene Expression Level

R language was used to calculate the correlation coefficient, the correlation between the expression level of *PgGTs* and 16 verified ginsenoside key enzymes was calculated, the expression level was analyzed and the heat map was drawn. IBM SPSS Statistics version 23.0 software was used to analyze the significance of key enzyme genes and *PgGTs* in the three modules, and BioLayout Express^3D^ version 3.3 software [58] was used to construct the co-expression network of *PgGT* genes and key enzyme genes (Table S2).

### 4.4. Culture of ABA-Induced Ginseng Hairy Roots

Ginseng hair roots were cultured in liquid medium without any hormone. After 20 days of culture, abscisic acid (ABA) was added to different culture bottles for further culture. The processing time presented 10 gradients: 0 h, 12 h, 18 h, 24 h, 36 h, 48 h, 60 h, 72 h, 96 h and 120 h. Ginseng hair roots without ABA treatment were used as the control. Samples were taken out at the end time point of the culture, and the dry weight and fresh weight of ginseng hair roots were measured and recorded. When weighing fresh weight, a small number of samples were selected and stored in a refrigerator at −80 °C for RNA extraction for fluorescence quantitative PCR to detect the expression levels of *PgGT* genes and key enzyme genes (primers are shown in Table 1). The remaining part was weighed as fresh weight, dried, dry weight data were recorded and saponins were extracted for preparation in follow-up experiments.

### 4.5. RNA Extraction and Fluorescence Quantitative PCR

The total RNA of ginseng was extracted via the Trizol method and reverse-transcribed into cDNA using a relevant kit. The key enzyme gene *ACT1* of the restriction reaction in the synthesis of saposides was selected as the internal reference gene, and the SYBR Premix Ex Taq TTM Ⅱ (Tli RNaseH Plus) fluorescence quantitative PCR kit was used for the subsequent fluorescence quantitative PCR reaction of each gene. The reaction system for fluorescence quantitative PCR was as follows: the first step was 95 °C, 10 min; the second step was 95 °C, 15 s; and the third step was 60 °C, 45 s, and 40 cycles were required: Solution curves were analyzed at 95 °C for 15 s, 60 °C for 45 s, 95 °C for 15 s and 60 °C for 15 s. The gene expression levels in the treated ginseng materials were detected by fluorescence quantitative PCR, and the samples were sampled in the sequence of the designed conditions. After three technical and biological replicates, the internal reference gene was normalized, and the final data results were calculated and mapped according to the 2^−ΔΔCT^ method.

### 4.6. Sequence and Expression Analysis of Desired Gene

The protein sequence of PgGT25-04 was input into ExPASy online software, and ProtParam Tool was used to analyze the physicochemical properties of the sequence. The secondary structure of the PgGT25-04 protein sequence was predicted using SOPMA, SWISS-MODEL was used to predict the tertiary structure of the PgGT25-04 protein sequence input, the prediction of subcellular location was performed via Wolf PSORT and PgGT25-04 transmembrane analysis was conducted in TMHMM. The phosphorylation sites of *PgGT25-04* were predicted using NetPhos online software. A phylogenetic tree of Arabidopsis Trihelix transcription factor and *PgGT25-04* was constructed using MEGA-X and adjacency methods. The expression levels of the *PgGT25-04* gene in the root of ginseng in different years, in 14 tissue parts and 42 farmer cultivars were extracted from the transcriptome database [57], and TBtools v1.108 [59] was used to draw a heat map.

### 4.7. Synthesis of PgGT25-04 Gene Silencing Sequence and Construction of Vector

The *PgGT25-04* gene silencing sequence was designed and sent to a biotechnology company (Shanghai, China) for synthesis. Using the primers PgGT25-04-RNAi-F: TCTAGATGTTTGGGATATGAAAATAGGAGT and PgGT25-04-RNAi-R: CCCGGGTGTTTGGGATATGAAAATAGGAG, PCR amplification was performed, and finally the product of the target fragment was recovered for use. The target fragment was inserted into the pBI121 expression vector at the appropriate restriction site to construct the *PgGT25-04* gene silencing expression vector.

### 4.8. Genetic Transformation of Adventitious Root of Ginseng

The recombinant plasmid was transferred into *Agrobacterium rhizogenes* C58C1 and stored for later use. The adventitious roots of ginseng were pretreated and cultured. The adventitious roots were cut into small segments of about 1 cm and cultured on MS solid medium containing hormones in inverted plates for 2 days. The next step was the co-culture of the bacterial solution and adventitious roots: first, the pre-cultured adventitious roots were cut into small segments and put into the bacterial solution for infection. The infected adventitious roots were then cultured on solid medium containing acetoeugenone. Finally, the sterilized explants were placed into the medium with cephalosporin for dark culture to observe the growth state of the explants [60].

### 4.9. Detection of Positive Materials

The primers corresponding to the samples that were transferred to target genes were designed for the positive test, the verification of target genes and expression vectors and the verification of RolC transferred to *Agrobacterium rhizogenes* in plants. The sequence of designed primers is shown in Table 1.

The single-root system of hair roots verified as positive was an expanded culture, and some of the samples were tested by means of fluorescence quantitative analysis to detect the expression levels of *PgGT25-04* and key enzyme genes. The other samples were dried to extract saponins.

### 4.10. Extraction of Ginsenosides

The sample was dried to a constant weight and ground into a fine powder. We then wrapped 0.5 g of the sample in filter paper and soaked it in a triangular bottle with methanol solution. The saponins were then extracted via Soxhlet extraction [61], filtered with an organic filter head and set aside.

### 4.11. Determination of Ginsenoside Using High-Performance Liquid Chromatography

The standard ginsenosides were weighed accurately, diluted and dissolved with methanol, and the resulting liquid solution was filtered through a 0.22 μM organic filter membrane for backup. The conditions for HPLC were as follows: water C18 column; the mobile phase included acetonitrile and water; the sample size was 20 μL and the mobile phase flow rate was 1 mL per minute. The column temperature was 35 °C and the detection wavelength was set to 203 nm. High-performance liquid phase gradient conditions are presented in Table 2.

### 4.12. Statistical Analysis of Data

The expression levels of key enzyme genes and *PgGT* genes were detected via fluorescence quantitative RT-PCR in ABA-treated ginseng materials with *PgGT25-04* gene overexpression. The 2^−ΔΔCT^ method was used for calculation and plotting. Ginsenoside content measured using the HPLC method was evaluated via variance analysis (ANOVA) and Student’s *t* test, and the mean standard error and significance difference of traits measured using transgenic strains were evaluated by comparison with the control group. The critical value was *p* ≤ 0.05.

## 5. Conclusions

In this study, firstly, WGCNA was performed on *PgGTs* to obtain three co-expressed gene modules. Then, correlation analysis was conducted between *PgGT* genes in the module and ginsenoside and key enzyme genes in ginsenoside synthesis, and interaction network analysis was conducted with key enzyme genes. Finally, a *PgGT25-04* gene negatively correlated with ginsenoside synthesis was screened. We found that *PgGT* genes respond to ABA signals, and *PgGT25-04* gene expression is spatiotemporally specific in ginseng. The *PgGT25-04* gene expression in the positive ginseng hair root system was successfully obtained, and the *PgGT25-04* gene expression of the positive ginseng hair root system was decreased, while the saponin content and key enzyme gene expression were increased. It was preliminarily determined that the *PgGT25-04* gene inhibited ginsenoside synthesis in *Panax ginseng*.

## Figures and Tables

**Table 1:** All primers required for this study.

Gene Primer Name	Primer Sequence
*pBI121-R*	CGCAGTTCAACGCTGACATC
*35S-F*	GACGCACAATCCCACTATCC
*RolC-F*	ATGGCTGAAGACGACTTGTGTTC
*RolC-R*	TTAGCCGATTGCAAACTT
*PgGT07-01-F*	ACCATCAATCTCATCCAAGC
*PgGT07-01-R*	CGGTAGCGTTTACGGAGT
*PgGT16-01-F*	GGATACCTCCGCACAGCC
*PgGT16-01-R*	ACCGGGGTAGGAAAGAGC
*PgGT16-02-F*	CCCTCGTCGAAGCCTACC
*PgGT16-02-R*	GGCGGAGCTTCTCCATTT
*PgGT28-08-F*	CTTTGGAAGCAGGAGACG
*PgGT28-08-R*	AAGCAGCAGCCTAGCAGT
*PgGT28-19-F*	TCTGGTCCCACAGGTGCT
*PgGT28-19-R*	AAACATGGGTCCAAGAAG
*PgGT25-04-F*	GAGCCTATGTTTGTTCAGC
*PgGT25-04-R*	GCTATCCCACTTGTCTTTG
*CYP716A52v2_3-F*	TGTTGATTGGAGGCCATGAC
*CYP716A52v2_3-R*	CCACTTCAATAATTCTCCTGCC
*DS_3-F*	CGGAACGATTGACACTATTCTGAC
*DS_3-R*	CTGACCCAATCATCGTGCTGT
*UGT71A27-F*	TGCGTCCGTCTATCCCTAAAG
*UGT71A27-R*	TGATGTCCTGTCCAAGAATCCTAC
*FPS_22-F*	GGATGATTATCTGGATTGCTTTGG
*FPS_22-R*	CAGTGCTTTTACTACCAACCAGGAG
*SE2_1-F*	GCAAGGGACTGTGACATCTCTG
*SE2_1-R*	TGCTGCCGACATTTCTTGG
*CYP137-F*	GGACAACGAGGCAGCACTT
*CYP137-R*	AAGTGAGCGACTCTGACATAGCG
*DS_1-F*	CGGAACGATTGACACTATTCTGAC
*DS_1-R*	CTGACCCAATCATCGTGCTGT
*SE2-4-F*	TAATCGTCATCGGAGTCGCC
*SE2-4-R*	ACCCGACCCCTTATCTGTGA
*ACT1-F*	TGGCATCACTTTCTACAACG
*ACT1-R*	TTTGTGTCATCTTCTCCCTGTT

**Table 2:** Mobile phase gradient conditions for high-performance liquid chromatography.

Time (min)	Acetonitrile (%)	Water (%)
0–40	18–21	82–79
40–42	21–26	79–74
42–46	26–32	74–68
46–66	32–33.5	68–66.5
66–71	33.5–38	66.5–62
71–86	38–65	62–35
86–91	65	35
91–96	65–85	35–15
96–103	85	15
103–105	85–18	15–82
105–106	18	82

## Data Availability

The sequences of the *PgGT* genes will be submitted to NCBI under BioProject RJNA302556.

## Supplementary Materials

The following are available online at https://www.mdpi.com/article/10.3390/plants12101980/s1, Figure S1: Effects of ABA stress on gene expression levels of key enzymes in ginsenoside synthesis. The X axis represents the duration of ABA treatment of ginseng hairy roots. The Y axis represents the relative expression level of genes in ginseng hairy roots; Figure S2: Changes in ginsenoside content in ginseng hairy root after ABA treatment; Figure S3: Detection of positive single-root plants. M: DL2000 marker; 1–3: blank control; 4–27: positive single-root plant; Table S1: *PgGT* genes in three modules by WGCNA; Table S2: The expressions of the *PgGT* genes and key enzyme genes in ginsenoside synthesis in 14 tissues, 42 cultivars’ roots and 4 aged roots (TPM).

## References

[1] Latchman D.S. (1997). Transcription factors: An overview. Int. J. Biochem. Cell Biol..

[2] Song L., Li W., Chen X. (2022). Transcription factor is not just a transcription factor. Trends Plant Sci..

[3] Sakuma Y., Liu Q., Dubouzet J.G., Abe H., Shinozaki K., Yamaguchi-Shinozaki K. (2002). DNA-binding specificity of the ERF/AP2 domain of Arabidopsis DREBs, transcription factors involved in dehydration- and cold-inducible gene expression. Biochem. Biophys. Res. Commun..

[4] Wang Z., Gong M., Liu Y., Xiong S., Wang M., Zhou J., Zhang Y. (2022). Towards a better understanding of TF-DNA binding prediction from genomic features. Comput. Biol. Med..

[5] Luo J.L., Zhao N., Lu C.M. (2012). Plant Trihelix transcription factors family. Yi Chuan.

[6] Dubos C., Stracke R., Grotewold E., Weisshaar B., Martin C., Lepiniec L. (2010). *MYB* transcription factors in *Arabidopsis*. Trends Plant Sci..

[7] Chu Y., Xiao S., Su H., Liao B., Zhang J., Xu J., Chen S. (2018). Genome-wide characterization and analysis of *bHLH* transcription factors in *Panax ginseng*. Acta Pharm. Sin. B..

[8] Feng K., Hou X.L., Xing G.M., Liu J.X., Duan A.Q., Xu Z.S., Li M.Y., Zhuang J., Xiong A.S. (2020). Advances in *AP2/ERF* super-family transcription factors in Plant. Crit. Rev. Biothchnol..

[9] Waseem M., Nkurikiyimfura O., Niyitanga S., Jakada B.H., Shaheen I., Aslam M.M. (2022). *GRAS* transcription factors emerging regulator in plants growth, development, and multiple stresses. Mol. Biol. Rep..

[10] Oda-Yamamizo C., Mitsuda N., Sakamoto S., Ogawa D., Ohme-Takagi M., Ohmiya A. (2016). The *NAC* transcription factor *ANAC046* is a positive regulator of chlorophyll degradation and senescence in *Arabidopsis* leaves. Sci. Rep..

[11] Zhu M., Bin J., Ding H., Pan D., Tian Q., Yang X., Wang L., Yue Y. (2022). Insights into the trihelix transcription factor responses to salt and other stresses in *Osmanthus fragrans*. BMC Genom..

[12] Courbier S., Hartman S. (2022). *WRKYs* work to limit root growth in response to shade. Plant Physiol..

[13] Cheng X., Xiong R., Yan H., Gao Y., Liu H., Wu M., Xiang Y. (2019). The trihelix family of transcription factors: Functional and evolutionary analysis in Moso bamboo (*Phyllostachys edulis*). BMC Plant Biol..

[14] Kaplan-Levy R.N., Brewer P.B., Quon T., Smyth D.R. (2012). The *trihelix* family of transcription factors—Light, stress and development. Trends Plant Sci..

[15] Nagano Y. (2000). Several features of the *GT*-factor *trihelix* domain resemble those of the Myb DNA-binding domain. Plant Physiol..

[16] Xie Z.M., Zou H.F., Lei G., Wei W., Zhou Q.Y., Niu C.F., Liao Y., Tian A.G., Ma B., Zhang W.K. (2009). Soybean *Trihelix* transcription factors *GmGT-2A* and *GmGT-2B* improve plant tolerance to abiotic stresses in transgenic *Arabidopsis*. PLoS ONE.

[17] Shibata M., Favero D.S., Takebayashi R., Takebayashi A., Kawamura A., Rymen B., Hosokawa Y., Sugimoto K. (2022). *Trihelix* transcription factors *GTL1* and *DF1* prevent aberrant root hair formation in an excess nutrient condition. New Phytol..

[18] Yang W., Hu J., Behera J.R., Kilaru A., Yuan Y., Zhai Y., Xu Y., Xie L., Zhang Y., Zhang Q. (2021). A tree peony *Trihelix* transcription factor *PrASIL1* represses seed oil Accumulation. Front. Plant Sci..

[19] Yu C., Song L., Song J., Ouyang B., Guo L., Shang L., Wang T., Li H., Zhang J., Ye Z. (2018). *ShCIGT*, a *Trihelix* family gene, mediates cold and drought tolerance by interacting with *SnRK1* in tomato. Plant Sci..

[20] Wang X.H., Li Q.T., Chen H.W., Zhang W.K., Ma B., Chen S.Y., Zhang J.S. (2014). *Trihelix* transcription factor *GT-4* mediates salt tolerance via interaction with *TEM2* in *Arabidopsis*. BMC Plant Biol..

[21] Zheng X., Liu H., Ji H., Wang Y., Dong B., Qiao Y., Liu M., Li X. (2016). The wheat *GT* factor *TaGT2L1D* negatively regulates drought tolerance and plant development. Sci. Rep..

[22] Feng C., Song X., Tang H. (2019). Molecular cloning and expression analysis of *GT-2*-like genes in strawberry. 3 Biotech.

[23] Luo J., Tang S., Mei F., Peng X., Li J., Li X., Yan X., Zeng X., Liu F., Wu Y. (2017). *BnSIP1-1*, a *Trihelix* family gene, mediates abiotic stress tolerance and ABA signaling in *Brassica napus*. Front. Plant Sci..

[24] Liu H.F., Zhang T.T., Liu Y.Q., Kang H., Rui L., Wang D.R., You C.X., Xue X.M., Wang X.F. (2023). Genome-wide analysis of the 6B-INTERACTING PROTEIN1 gene family with functional characterization of MdSIP1-2 in *Malus domestica*. Plant Physiol. Biochem..

[25] Schroeder J.I., Kwak J.M., Allen G.J. (2001). Guard cell abscisic acid signalling and engineering drought hardiness in plants. Nature.

[26] Gagné S., Cluzet S., Mérillon J., Gény L. (2011). ABA initiates anthocyanin production in grape cell cultures. J. Plant Growth Regul..

[27] Mansouri H., Asrar Z., Mehrabani M. (2009). Effects of gibberellic acid on primary terpenoids and delta-tetrahydrocannabinol in *Cannabis sativa* at flowering stage. J. Integr. Plant Biol..

[28] Yang D., Sheng D., Duan Q., Liang X., Liang Z., Liu Y. (2012). PEG and ABA trigger the burst of reactive oxygen species to increase tanshinone production in *Salvia miltiorrhiza* hairy roots. J. Plant Growth Regul..

[29] Kochan E., Balcerczak E., Szymczyk P., Sienkiewicz M., Zielińska-Bliźniewska H., Szymańska G. (2019). Abscisic acid regulates the 3-Hydroxy-3-methylglutaryl CoA reductase gene promoter and ginsenoside production in *Panax quinquefolium* hairy root cultures. Int. J. Mol. Sci..

[30] Mancuso C., Santangelo R. (2017). *Panax ginseng* and *Panax quinquefolius*: From pharmacology to toxicology. Food Chem. Toxicol..

[31] Kiefer D., Pantuso T. (2003). *Panax ginseng*. Am. Fam. Physician.

[32] Guo M., Shao S., Wang D., Zhao D., Wang M. (2021). Recent progress in polysaccharides from *Panax ginseng* C. A. Meyer. Food Funct..

[33] Hou M., Wang R., Zhao S., Wang Z. (2021). Ginsenosides in *Panax* genus and their biosynthesis. Acta Pharm. Sin. B.

[34] Wang H.P., Liu Y., Chen C., Xiao H.B. (2017). Screening specific biomarkers of herbs using a metabolomics approach: A case study of *Panax ginseng*. Sci. Rep..

[35] Yang J.L., Hu Z.F., Zhang T.T., Gu A.D., Gong T., Zhu P. (2018). Progress on the studies of the key enzymes of ginsenoside biosynthesis. Molecules.

[36] Liu M., Pan Z., Yu J., Zhu L., Zhao M., Wang Y., Chen P., Liu C., Hu J., Liu T. (2022). Transcriptome-wide characterization, evolutionary analysis, and expression pattern analysis of the *NF-Y* transcription factor gene family and salt stress response in *Panax ginseng*. BMC Plant Biol..

[37] Liu M., Li K., Sheng S., Wang M., Hua P., Wang Y., Chen P., Wang K., Zhao M., Wang Y. (2022). Transcriptome analysis of *MYB* transcription factors family and *PgMYB* genes involved in salt stress resistance in *Panax ginseng*. BMC Plant Biol..

[38] Zhu L., Zhao M., Chen M., Li L., Jiang Y., Liu S., Jiang Y., Wang K., Wang Y., Sun C. (2020). The *bHLH* gene family and its response to saline stress in Jilin ginseng, *Panax ginseng* C.A. Meyer. Mol. Genet. Genom..

[39] Chen J., Zhou Y., Zhang Q., Liu Q., Li L., Sun C., Wang K., Wang Y., Zhao M., Li H. (2020). Structural variation, functional differentiation and expression characteristics of the *AP2/ERF* gene family and its response to cold stress and methyl jasmonate in *Panax ginseng* C.A. Meyer. PLoS ONE.

[40] Wang N., Wang K., Li S., Jiang Y., Li L., Zhao M., Jiang Y., Zhu L., Wang Y., Su Y. (2020). Transcriptome-wide identification, evolutionary analysis, and GA stress response of the *GRAS* gene family in *Panax ginseng* C. A. Meyer. Plants.

[41] Liu Q., Sun C., Han J., Li L., Wang K., Wang Y., Chen J., Zhao M., Wang Y., Zhang M. (2020). Identification, characterization and functional differentiation of the *NAC* gene family and its roles in response to cold stress in ginseng, *Panax ginseng* C.A. Meyer. PLoS ONE.

[42] Di P., Wang P., Yan M., Han P., Huang X., Yin L., Yan Y., Xu Y., Wang Y. (2021). Genome-wide characterization and analysis of *WRKY* transcription factors in *Panax ginseng*. BMC Genom..

[43] Liu C., Wang K., Yun Z., Liu W., Zhao M., Wang Y., Hu J., Liu T., Wang N., Wang Y. (2023). Functional study of *PgGRAS68-01* gene involved in the regulation of ginsenoside biosynthesis in *Panax ginseng*. Int. J. Mol. Sci..

[44] Li J., Chen X., Zhou X., Huang H., Wu D., Shao J., Zhan R., Chen L. (2021). Identification of trihelix transcription factors in *Pogostemon cablin* reveals PatGT-1 negatively regulates patchoulol biosynthesis. Ind. Crop Prod..

[45] Jiao H., Hua Z., Zhou J., Hu J., Zhao Y., Wang Y., Yuan Y., Huang L. (2023). Genome-wide analysis of Panax *MADS*-box genes reveals role of *PgMADS41* and *PgMADS44* in modulation of root development and ginsenoside synthesis. Int. J. Biol. Macromol..

[46] Langfelder P., Horvath S. (2008). WGCNA: An R package for weighted correlation network analysis. BMC Bioinform..

[47] Ma J.J., Chen X., Song Y.T., Zhang G.F., Zhou X.Q., Que S.P., Mao F., Pervaiz T., Lin J.X., Li Y. (2021). MADS-box transcription factors *MADS11* and *DAL1* interact to mediate the vegetative-to-reproductive transition in pine. Plant Physiol..

[48] Cui Y.X., Xu Z.C., Chen X.L., Nie L.P., Wu L.W., Wang Y., Song J.Y., Yao H. (2020). Genome-wide identification of abscisic acid (ABA) receptor pyrabactin resistance 1-like protein (PYL) family members and expression analysis of *PYL* genes in response to different concentrations of ABA stress in *Glycyrrhiza uralensis*. Chin. J. Nat. Med..

[49] Bai G., Xie H., Yao H., Li F., Chen X., Zhang Y., Xiao B., Yang J., Li Y., Yang D.H. (2019). Genome-wide identification and characterization of ABA receptor *PYL/RCAR* gene family reveals evolution and roles in drought stress in *Nicotiana tabacum*. BMC Genom..

[50] Wang Y.G., Yu H.Q., Zhang Y.Y., Lai C.X., She Y.H., Li W.C., Fu F.L. (2014). Interaction between abscisic acid receptor PYL3 and protein phosphatase type 2C in response to ABA signaling in maize. Gene.

[51] Hung Y.H., Slotkin R.K. (2021). The initiation of RNA interference (RNAi) in plants. Curr. Opin. Plant Biol..

[52] Bai X., Huang X., Tian S., Peng H., Zhan G., Goher F., Guo J., Kang Z., Guo J. (2021). RNAi-mediated stable silencing of *TaCSN5* confers broad-spectrum resistance to *Puccinia striiformis* f. sp. tritici. Mol. Plant Pathol..

[53] Liu S., Geng S., Li A., Mao Y., Mao L. (2021). RNAi technology for plant protection and its application in wheat. aBIOTECH.

[54] Lu C., Zhao S., Wei G., Zhao H., Qu Q. (2017). Functional regulation of ginsenoside biosynthesis by RNA interferences of a UDP-glycosyltransferase gene in *Panax ginseng* and *Panax quinquefolius*. Plant Physiol. Biochem..

[55] Zhao C., Xu T., Liang Y., Zhao S., Ren L., Wang Q., Dou B. (2015). Functional analysis of β-amyrin synthase gene in ginsenoside biosynthesis by RNA interference. Plant Cell Rep..

[56] García-Cano E., Magori S., Sun Q., Ding Z., Lazarowitz S.G., Citovsky V. (2015). Interaction of *Arabidopsis trihelix*-domain transcription factors *VFP3* and *VFP5* with Agrobacterium Virulence protein VirF. PLoS ONE.

[57] Wang K., Jiang S., Sun C., Lin Y., Yin R., Wang Y., Zhang M. (2015). The spatial and temporal transcriptomic landscapes of ginseng, *Panax ginseng* C. A. Meyer. Sci. Rep..

[58] Wright D.W., Angus T., Enright A.J., Freeman T.C. (2014). Visualisation of bioPAX networks using BioLayout Express (3D). F1000Research.

[59] Chen C., Chen H., Zhang Y., Thomas H.R., Frank M.H., He Y., Xia R. (2020). TBtools: An integrative toolkit developed for interactive analyses of big biological data. Mol. Plant.

[60] Kim Y.K., Kim Y.B., Uddin M.R., Lee S., Kim S.U., Park S.U. (2014). Enhanced triterpene accumulation in *Panax ginseng* hairy roots overexpressing mevalonate-5-pyrophosphate decarboxylase and farnesyl pyrophosphate synthase. ACS Synth. Biol..

[61] Sander L.C. (2017). Soxhlet extractions. J. Res. Natl. Inst. Stan.

