# Supplementary material for: Transcriptome-Wide Integrated Analysis of the PgGT25-04 Gene in Controlling Ginsenoside Biosynthesis in Panax ginseng
Source: Plants (Basel). 2023 May 15;12(10):1980. doi: 10.3390/plants12101980 (PMC10224475; doi:10.3390/plants12101980)
Supplement: Supplementary file 1 [file plants-12-01980-s001.zip › Figure S1.pptx]

## Slide 1
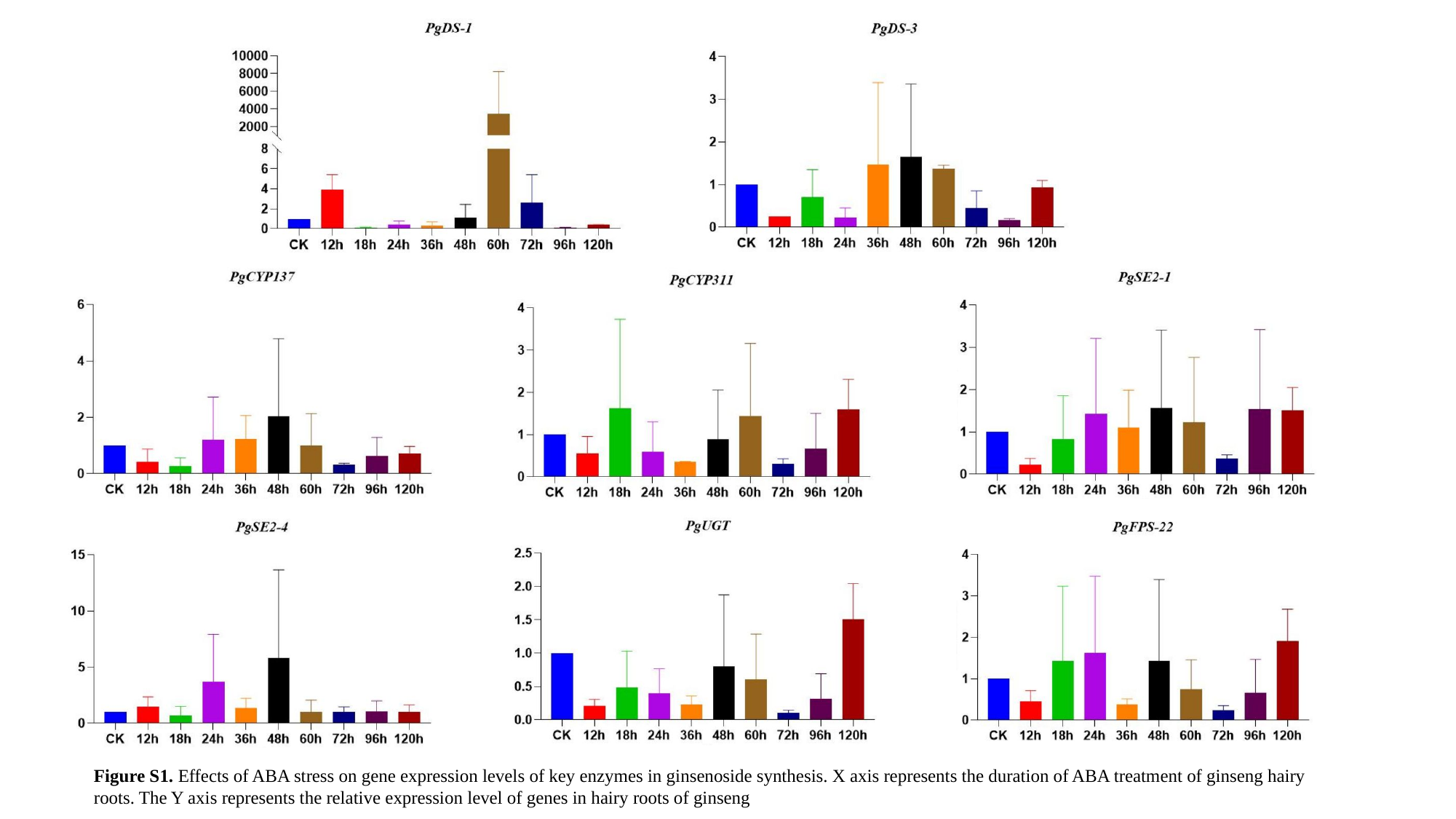

Figure S1. Effects of ABA stress on gene expression levels of key enzymes in ginsenoside synthesis. X axis represents the duration of ABA treatment of ginseng hairy roots. The Y axis represents the relative expression level of genes in hairy roots of ginseng
