# Supplementary material for: Transcriptome-Wide Integrated Analysis of the PgGT25-04 Gene in Controlling Ginsenoside Biosynthesis in Panax ginseng
Source: Plants (Basel). 2023 May 15;12(10):1980. doi: 10.3390/plants12101980 (PMC10224475; doi:10.3390/plants12101980)
Supplement: Supplementary file 1 [file plants-12-01980-s001.zip › Figure S2.pptx]

## Slide 1
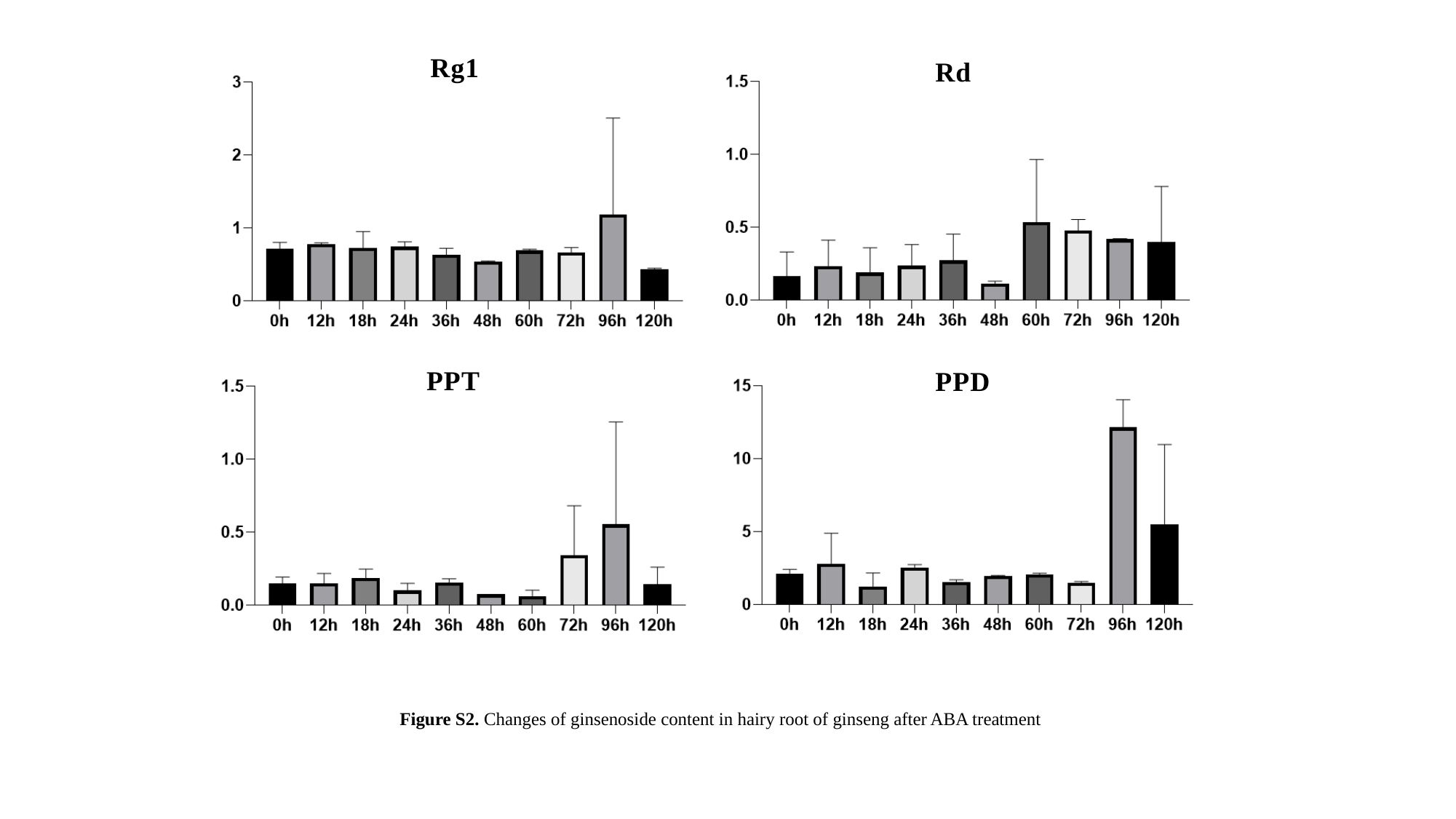

Rg1
Rd
PPT
PPD
Figure S2. Changes of ginsenoside content in hairy root of ginseng after ABA treatment
