# Supplementary material for: Transcriptome-Wide Integrated Analysis of the PgGT25-04 Gene in Controlling Ginsenoside Biosynthesis in Panax ginseng
Source: Plants (Basel). 2023 May 15;12(10):1980. doi: 10.3390/plants12101980 (PMC10224475; doi:10.3390/plants12101980)
Supplement: Supplementary file 1 [file plants-12-01980-s001.zip › Figure S3.pptx]

## Slide 1
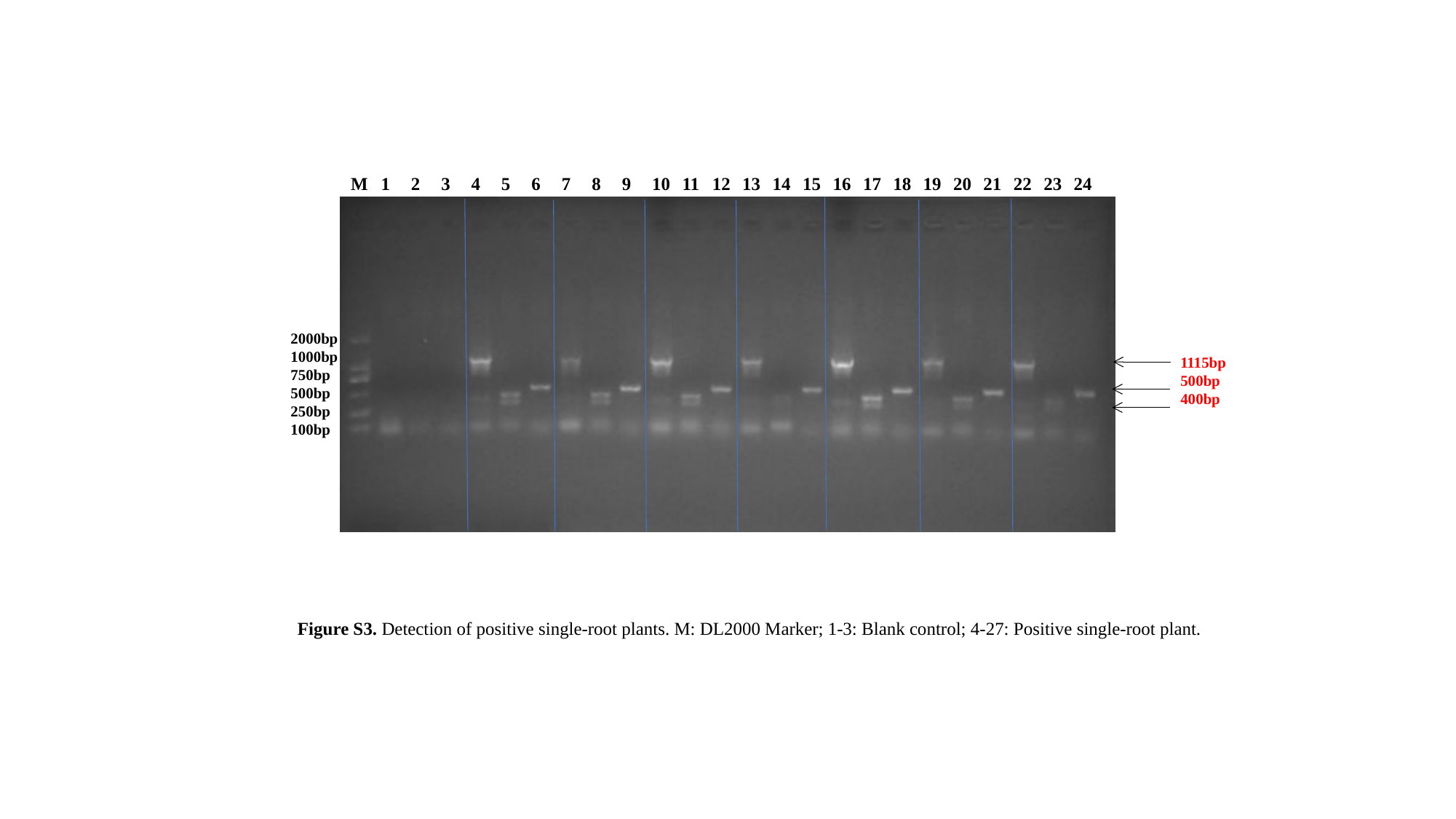

M
1
2
3
4
5
6
7
8
9
10
11
12
13
14
15
16
17
18
19
20
21
22
23
24
2000bp
1000bp
750bp
500bp
250bp
100bp
1115bp
500bp
400bp
Figure S3. Detection of positive single-root plants. M: DL2000 Marker; 1-3: Blank control; 4-27: Positive single-root plant.
